# Gene-Gene Interaction Analysis for the Survival Phenotype Based on the Kaplan-Meier Median Estimate

**DOI:** 10.1155/2020/5282345

**Published:** 2020-05-09

**Authors:** Mira Park, Jung Wun Lee, Taesung Park, SeungYeoun Lee

**Affiliations:** ^1^Department of Preventive Medicine, Eulji University, Daejeon, Republic of Korea; ^2^Department of Statistics, University of Connecticut, Storrs, Connecticut, USA; ^3^Department of Statistics, Seoul National University, Seoul, Republic of Korea; ^4^Department of Mathematics and Statistics, Sejong University, Seoul, Republic of Korea

## Abstract

In this study, we propose a simple and computationally efficient method based on the multifactor dimensional reduction algorithm to identify gene-gene interactions associated with the survival phenotype. The proposed method, referred to as KM-MDR, uses the Kaplan-Meier median survival time as a classifier. The KM-MDR method classifies multilocus genotypes into a binary attribute for high- or low-risk groups using median survival time and replaces balanced accuracy with log-rank test statistics as a score to determine the best model. Through intensive simulation studies, we compared the power of KM-MDR with that of Surv-MDR, Cox-MDR, and AFT-MDR. It was found that KM-MDR has a similar power to that of Surv-MDR, with less computing time, and has comparable power to that of Cox-MDR and AFT-MDR, even when there is a covariate effect. Furthermore, we apply KM-MDR to a real dataset of ovarian cancer patients from The Cancer Genome Atlas (TCGA).

## 1. Introduction

In this era of precision medicine, one of the main goals of human genetics is to understand the biological relationship between diseases and their treatment so that each patient receives the best treatment based on his or her genetic and/or environmental exposures [1]. To achieve this goal, it is necessary to identify the genetic or environmental factors associated with various diseases. With the recent development of high-throughput technologies, information has been made available on a large number of genetic variants, such as single-nucleotide polymorphisms (SNPs), and genome-wide association studies (GWAS) have successfully discovered many susceptibility genes associated with various diseases. As of June 20, 2019, the GWAS catalog contains 4,038 publications and 118,709 associations (http://http://www.ebi.ac.uk/gwas). However, the SNPs identified in GWAS have limitations in explaining missing heritability. One possible way to overcome this problem is to identify the effects of gene-gene interactions and/or gene-environmental interactions on complex diseases [2].

The multifactor dimensionality reduction (MDR) method has been widely applied to identify gene-gene interactions (GGIs) [3]. The main idea of MDR is to reduce the dimensionality of multilocus information into one-dimensional binary attributes by pooling multilocus genotypes into either a high-risk group or a low-risk group. Then, cross-validation is used to evaluate the ability of the generated binary variables to classify and predict outcomes. Since its first introduction for binary traits, numerous studies have modified and extended the original MDR method [4]. Log-linear model-based MDR (LM-MDR) improved factor combinations using log-linear models and reclassifications of risk [5], whereas odds-ratio-based MDR (OR-MDR) used odds ratios instead of naive classifiers [6]. MDR methods for imbalanced data [7] and incomplete data [8] have been also developed. For continuous traits, the generalized MDR (GMDR) method was proposed to extend the MDR algorithm to be applicable to both dichotomous and continuous phenotypes [9] by using the residual score of the generalized linear model as a new classifier. The model-based MDR (MB-MDR) method was suggested as a flexible way to consider covariates or continuous traits [10] based on a regression model. In addition, quantitative MDR (QMDR) was proposed as a simple and efficient way to treat continuous traits [11]. QMDR classifies multilocus genotypes into either a high- or low-risk group by comparing the mean value of each multilocus genotype to the overall mean and then uses the *t* test statistic as a score to determine the best model. As multivariate extensions of QMDR, multi-QMDR and multi-CMDR based on Hotelling's T^2^ statistic were also proposed [12, 13].

However, there have been relatively few attempts to develop statistical methods to identify GGIs in the context of survival analysis. In cancer research, survival time has served as an important phenotype in association studies that have investigated genetic factors as well as environmental and clinical variables. Therefore, it is more informative to consider the censored survival phenotype than simply to treat the phenotype as a binary variable of death or survival. The Surv-MDR method was first proposed to handle censored time-to-event data, in which log-rank test statistics are calculated to compare the survival time between samples with and without the specific genotype combination for each multilocus genotype combination [14]. If the log-rank statistic is positive, the corresponding genotype is labeled as high-risk, and if not, it is classified as low-risk. Once all genotypes are classified as high- or low-risk, a new binary attribute is defined by pooling the high-risk genotype combinations into one group and the low-risk genotype combinations into another group. Next, the log-rank test is applied to compare these two survival curves, and the square of this log-rank statistic is used as the score to choose the best model, while the remaining cross-validation procedure is the same as done in the traditional MDR method [4, 14]. Although Surv-MDR uses a computationally simple log-rank test, *3^q^* log-rank test statistics should be calculated for each SNP combination for a *q*^*t*h^-order interaction, which requires intensive computing time.

Additionally, as extended versions of GMDR for the survival phenotype, both Cox-MDR and accelerated failure time MDR (AFT-MDR) were proposed as methods in which the residual score of the generalized linear model is replaced with the appropriate measures corresponding to the survival models [15, 16]. In other words, Cox-MDR uses the martingale residual of a Cox model, and AFT-MDR uses the standardized residual from an accelerated failure time model to classify multilocus genotypes into high- and low-risk groups. Both Cox-MDR and AFT-MDR have the major advantage of adjusting for the covariate effect because these methods are based on regression models such as the Cox model and the AFT model. The cross-validation procedure is the same as the traditional MDR and uses balanced accuracy.

In this study, we propose a simple and computationally efficient method using the Kaplan-Meier median survival time, referred to as KM-MDR. This method conceptually extends the key idea of QMDR to the survival phenotype by replacing the mean value and the *t* test statistic with the median survival time and a log-rank test statistic, respectively. Since survival time is commonly censored and has a skewed distribution, the median survival time is more useful and popular statistic to make statistical inferences in survival analysis. KM-MDR uses the Kaplan-Meier median survival time to classify multilocus genotypes into the binary attribute of high- and low-risk groups. Once all multigenotypes are classified, we pool the high-risk genotype combinations into one group and the low-risk combinations into another group. Next, the log-rank test is applied to these two groups to choose the best model among all possible SNP combinations. Since the log-rank test is model-free, KM-MDR is nonparametric, as is Surv-MDR. However, KM-MDR is more efficient in the sense that the computing time for the median survival time is shorter than that for the log-rank test. Furthermore, Surv-MDR uses all data repeatedly by comparing the survival time between samples with and without a specific genotype combination, while KM-MDR uses all data once by comparing the median survival time of a specific genotype combination with the overall median survival time.

In Section 2 we detail the algorithm of the proposed KM-MDR method and present simulation results to compare the performance of KM-MDR with that of Surv-MDR, Cox-MDR, and AFT-MDR in Section 3 We apply the KM-MDR to a real dataset of ovarian cancer patients from The Cancer Genome Atlas (TCGA) in Section 4 and a short discussion is presented in Section 5.

## 2. Methods

As mentioned in the previous section, we propose a simple and computationally efficient method, KM-MDR, as an extension of QMDR to handle survival phenotypes. Instead of using the mean value of the quantitative trait as done in QMDR, we compare the Kaplan-Meier median survival time of each multilocus genotype combination to the overall median survival time. In addition, a log-rank test statistic is used instead of the *t* test statistic as a criterion for finding the best model. To determine the *q*-loci that provides the best model overall, we use 10-fold cross-validation as done in QMDR as follows.

**Step 1 .**

Divide the dataset by 10-folds.

- Suppose that we have *p* SNPs in the dataset and select *q* SNPs from *p* SNPs to consider a *q*-way interaction. For 10-fold cross-validations, the samples are randomly split into 10 subgroups of equal size. Then, 9/10 sets of samples are taken as the training dataset and the remaining sample is used as a testing dataset to evaluate the model.

**Step 2 .**

Classify multilocus genotypes into high- and low-risk groups by the Kaplan-Meier median survival time.

- For a *q*-way interaction, we construct multilocus genotype combinations defined by the *q* SNPs.
- Suppose that there are distinct and ordered death times, 0 ≤ *t*_1_ ≤ *t*_2_≤⋯≤*t*_*d*_, (*d* ≤ *n*) for the *n* individuals in the sample. First, we calculate the Kaplan-Meier median survival time, *t*_*m*_, from the overall sample, which is the smallest time *t* at which $\hat{S}\left(t\right)\leq 0.5$, where $\hat{S}\left(\text{t}\right)$ is a Kaplan-Meier estimate of the survival function, *S*(*t*), which is defined as $\begin{array}{c} \hat{S}\left(t\right)=\prod_{i:t_{i}\leq t}\;\left(1-d_{i}/n_{i}\right) \end{array}$. Here, *d*_*i*_ and *n*_*i*_ denote the number of events and the number of individuals at risk at time *t*_*i*_. Similarly, we calculate the median survival time, $\begin{array}{c} t_{mj} \end{array}$, for each cell having the *j*^*th*^ genotype combination, (*j* = 1, ⋯, 3^*q*^) in the training set.
- We compare $\begin{array}{c} t_{mj} \end{array}$ to $\begin{array}{c} t_{m} \end{array}$ and classify each cell into either the high-risk group if *t*_*mj*_ < *t*_*m*_, or the low-risk group, otherwise.
- If the median survival time is not available due to heavy censoring or the sparsity of the sample for a specific genotype combination, we make use of a complementary sample corresponding to this specific cell to estimate the median survival time. Here, this complementary sample is a pooled sample excluding the sample with the specific genotype combination. Since the complementary sample is made up of (3^*q*^ − 1) cells, it may be large enough to have the median survival time. If the median survival time for the complementary sample is larger than the overall median survival time, then this pooled sample is considered as the low-risk group. Thus, the sample with the specific genotype has smaller median survival time, which leads to be classified as the high-risk group.

**Step 3 .**

Calculate the log-rank test statistic to find the *q*-way best model.

- Once all genotype combinations are classified into high- and low-risk groups, we pool the high-risk genotype combinations into one group, “H,” and the low-risk combinations into another group, “L.” For the training set, the log-rank test statistic is calculated to test the equivalence of the two survival curves for the H and L groups as follows: Let *d*_*li*_ and *n*_*li*_ be the observed number of events and the number of individuals at risk in the *l*^*th*^  group (*l* = 1, 2) at time *t*_*i*_ and let *d*_*i*_ = *d*_1*i*_ + *d*_2*i*_  and *n*_*i*_ = *n*_1*i*_ + *n*_2*i*_ be the number of events and the number of at risk in the combined sample at *t*_*i*_. Then, the log-rank statistic is defined as Z = ∑_*i*=1_^*D*^(*d*_1*i*_ − *E*_1*i*_)/∑_*i*=1_^*D*^*V*_*i*_, where *E*_1*i*_ = *d*_*i*_(*n*_1*i*_/*n*_*i*_) and *V*_*i*_ = (*n*_*i*_ − *d*_*i*_)*d*_*i*_*n*_1*i*_*n*_2*i*_/(*n*_*i*_ − 1)*n*_*i*_^2^.
- We take the square of the log-rank test statistic as the score to characterize the relationship between survival time and gene-gene interaction. We use the training score from the log-rank test to select the best SNP pairs with the maximum training score among all possible SNP pairs. Next, we predict high- and low-risk status in the testing set corresponding to the training set.
- We repeated the above procedure 10 times so that each partition is included in the testing dataset once. Then, we calculate a testing score by using the log-rank test for all possible 10 testing sets. In addition, the number of times is counted that each *q*-way model chosen from the training datasets is identified as the best model; this is called cross-validation consistency (CVC). Then we select the best *q*-way interaction model with the maximum CVC.

**Step 4 .**

Find the overall best model.

- Repeat Step 3 above for all possible *q*-way models of interest (*q* = 1, 2, ⋯, *p*). Then we select the best model that has the maximum testing score and the highest CVC. The latter is used as a tie-break. If both statistics are tied, then the more parsimonious model is selected as the overall best model as done in [11].

As described in steps 1-4 above, any higher-order interaction model can be easily considered by the algorithm of MDR through cross-validation procedure. If possible, this cross-validation can be repeated to avoid the fluctuations due to chance divisions of the data. Furthermore, we determine the statistical significance of the selected model by comparing the testing score from observed data to the distribution of the testing scores under the null hypothesis of no association derived empirically from 1000 permutations. The null hypothesis is rejected if this empirical *p*-value is less than the significance level.

**Figure 1:**
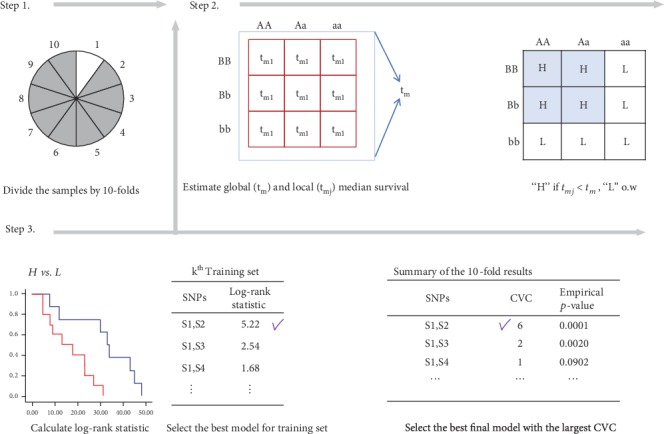
The KM-MDR algorithm for a 2-way interaction model with 10-fold cross-validation.

Here, we display the algorithm of KM-MDR for 2-way interaction model in Figure 1.

## 3. Results

### 3.1. Simulation Study

In this section, we present the results of simulation studies comparing the performance of the proposed KM-MDR method with that of Surv-MDR, Cox-MDR, and AFT-MDR. As discussed in the previous section, both Surv-MDR and KM-MDR are nonparametric methods that cannot adjust for covariate effects, while both Cox-MDR and AFT-MDR are based on a regression model and can adjust for covariate effects. We consider two different scenarios for the power comparison: the first one is a model without a covariate effect, and the second is a model with a covariate effect.

For the simulation study, we considered 10 SNPs that satisfied the assumption of Hardy–Weinberg equilibrium and linkage equilibrium. Among them, we let only two SNPs (SNP1 and SNP2) have causal SNP interactions with the survival time. We generated datasets based on different penetrance functions that define a probabilistic relationship between the outcome and SNPs in which the outcome was dependent on genotypes from two loci in the absence of any marginal effects [8]. These models were distributed across seven different heritability values (0.01, 0.025, 0.05, 0.1, 0.2, 0.3, and 0.4) and two different minor allele frequencies (0.2 and 0.4). A total of five models for each of the 14 heritability minor allele frequency combinations were generated for a total of 70 epistatic models, which are given in detail in [8].

Let *f*_*ij*_ be an element from the *i*^th^ row and the *j*^th^ column of a penetrance function for the two causal SNPs (SNP1 and SNP2), defined as.

(1) $$\begin{array}{c} f_{ij}=P\left(high\;risk\;\;|\;\;SNP1=i,SNP2=j\right). \end{array}$$

We sampled 400 patients from each of the 70 penetrance models to generate one simulated dataset and repeated this process 100 times to generate 100 datasets for each model. Then we simulated the survival times using both a Cox model and an AFT model.

Let *x* be an indicator variable with a value of 1 for high-risk patients and 0 for low-risk patients, and let *z* be an adjusting covariate generated from *N* (0,1). For a Cox model, given as *λ*(*t* | *x*, *z*) = *λ*_0_(*t*)exp(*xβ* + *zγ*), we set *β* = 1.2 and *γ* = 0 or 1. The baseline hazard function, *λ*_0_(t), was assumed to follow a Weibull distribution with a shape parameter of 5 and a scale parameter of 2, and the censoring time was generated from a uniform distribution *U* (0,4) as in [14]. For an AFT model given as log(*T*) = *μ* + *xβ* + *zγ* + *σε*, we set *μ* = 0, *β* = −1.0, and *γ* = 0 or 1. For the error distribution, we assumed that *ε* followed the normal distribution and that *σ* = 1.0. We also considered four different censoring fractions, (C): 0.0, 0.1, 0.3, and 0.5.

In all, 70 (penetrance model) × 2 (survival model) × 2 (covariate effect) × 4 (censoring fraction) = 1,120 different simulated datasets were repeated 100 times. The power was estimated as the percentage of times that each method correctly chose the causal SNP pairs (SNP1 and SNP2) as the best model among all possible two-way interaction models out of each set of 100 datasets. Parallel to the definition of this power, the Type I error of MDR has estimated the percentage of times that each method chose a certain SNP pair as the best model among all possible two-way interaction models under the null hypothesis. We generated 1000 null models with 8 non-causal SNPs for the Type I error. Then the selection rate of each SNP pair under the null model is $1/\left(\begin{array}{c} 8\\ 2 \end{array}\right)=1/28=0.03577$. We considered five different minor allele frequencies (0.05, 0.1, 0.2, 0.3, 0.4) and four different censoring fractions (0.0, 0.1, 0.3, 0.5). As shown in Table 1, the Type 1 error was well controlled (<0.03577) and seemed to be rather conservative.

**Figure 2:**
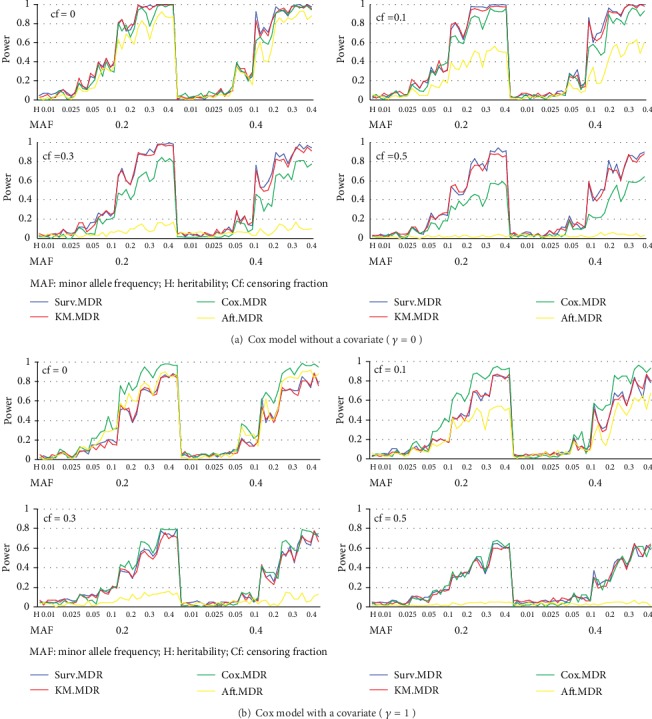
Power comparison of KM-MDR, Surv-MDR, Cox-MDR, and AFT-MDR for a Cox model (a) without a covariate (*γ* = 0) and (b) with a covariate (*γ* = 1).

**Figure 3:**
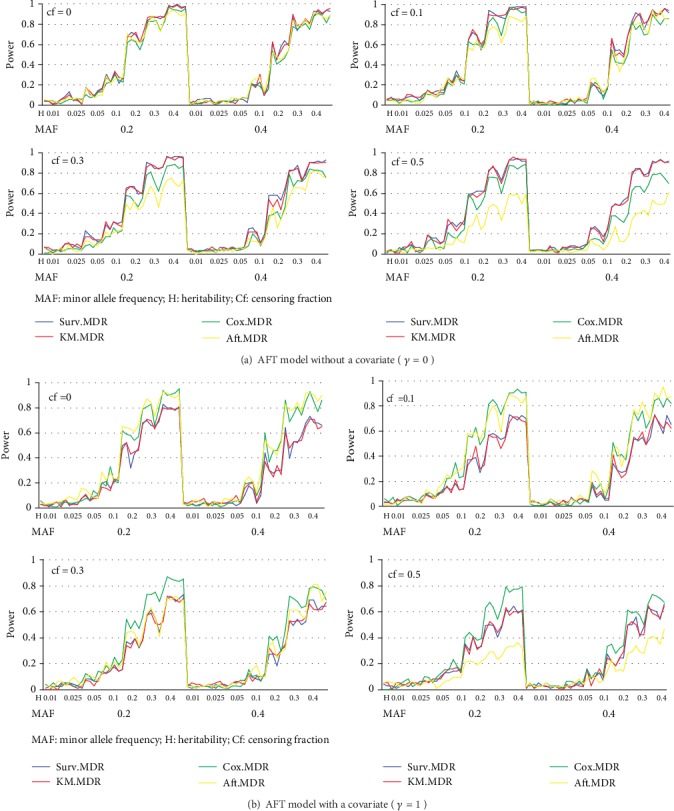
Power comparison of KM-MDR, Surv-MDR, Cox-MDR, and AFT-MDR for an AFT model (a) without a covariate (*γ* = 0) and (b) with a covariate (*γ* = 1).

For the power comparison, Figure 2 displays the power obtained from a Cox model and Figure 3 shows the power obtained from an AFT model. In each Figure, eight different plots are shown according to combinations of the presence of a covariate effect (*γ* = 0, 1) and the four different censoring fractions. In each plot, four different powers of KM-MDR, Surv-MDR, Cox-MDR, and AFT-MDR are overlaid with different types of lines across 70 models by ordering the combination of MAF and heritability, where 5 different models are given for each combination of MAF and heritability (2 × 7 × 5 = 70 models).

First of all, a common property of all methods is that the power tended to increase as heritability increased, but decreased as the censoring fraction increased. In addition, the power seemed to be greater with MAF = 0.2 than with MAF = 0.4. It is also noted that both KM-MDR and Surv-MDR have almost the same power across all cases.

The power of KM-MDR was greater than that of both Cox-MDR and AFT-MDR when there was no covariate effect, while KM-MDR had a somewhat lower power than either Cox-MDR or AFT-MDR when there was a covariate effect. However, the power of KM-MDR was comparable with that of Cox-MDR when the censoring fraction was greater than 0.3, although there was a covariate effect. The power of KM-MDR also seemed to be robust to the censoring fraction, similarly to Cox-MDR. However, the power of AFT-MDR was very sensitive to the censoring fraction because it was very low under a Cox model and had the lowest power even under an AFT model when the censoring fraction was greater than 0.3.

In summary, KM-MDR had a power almost identical to that of Surv-MDR across all cases and outperformed Cox-MDR under a model without a covariate effect. In addition, KM-MDR had reasonable power even under heavy censoring and showed comparable power to that of Cox-MDR even when there was a covariate effect.

### 3.2. Real Data Analysis

We illustrate the proposed method by analyzing ovarian cancer patient data from The Cancer Genome Atlas (TCGA) at https://gdc.xenahubs.net. This dataset consists of 433 ovarian cancer patients with 565 SNPs, in which 207 patients died from ovarian cancer, whereas 226 patients were censored. We first analyzed this data with all 565 SNPs using both KM-MDR and Surv-MDR and compared these two sets of results. In addition, 20 of the 565 SNPs showed a significant main effect in a single SNP analysis using a Cox regression model. In order to distinguish a true multiplicative model from an additive effect model, we also reanalyzed this dataset with 545 SNPs after removing the 20 SNPs that had significant main effects. We present the 20 SNPs with a significant main effect when we fit a Cox regression model with only each single SNP in Table 2. We applied 10-fold cross-validation by keeping the censoring fraction the same for alandomly split samples.

For simplicity of comparison, we display the top three two-way interaction models identified by KM-MDR and Surv-MDR with all 565 SNPs and with 545 SNPs, respectively, in Table 3. Similarly, we display the top three two-way interaction models identified by Surv-MDR in Table 4. We implemented 10-fold cross-validation and selected the best pair of SNPs by comparing both the CVC and testing scores. As displayed in Figure 1, the CVC was used for selecting the best pair, and the testing score was used as a tie-breaker. However, the maximum value of CVC only identified one of the pairs in 10-fold cross-validation, and thus the testing score was used to select the top three pairs. In Tables 3 and 4, the training score is the average of 10 training scores across 10-fold cross-validation, whereas the testing score is a log-rank test statistic calculated from the whole dataset by combining all 10 disjoint testing sets. We also conducted 1000 permutations to obtain a *p* value to check the statistical significance of the selected model. Each table provides the selected model, training score, testing score, CVC, and permutation *p* value.

Comparing the results shown in Tables 3 and 4, four SNP pairs overlapped between KM-MDR and Surv-MDR, and their testing scores were very similar. This result is consistent with the finding from the simulation studies that the power of both KM-MDR and Surv-MDR is almost the same. When all 565 SNPs were included in the analysis, only one pair (rs143372586 and rs61937629) included the rs143372586 SNP, which had a significant main effect, as shown in Table 2. This pair was identified as one of the top three pairs by both KM-MDR and Surv-MDR but did not appear in the analysis with 545 SNPs because the rs143372586 SNP was excluded. It can also be noted that all the permutation *p* values were significant for both KM-MDR and Surv-MDR.

**Figure 4:**
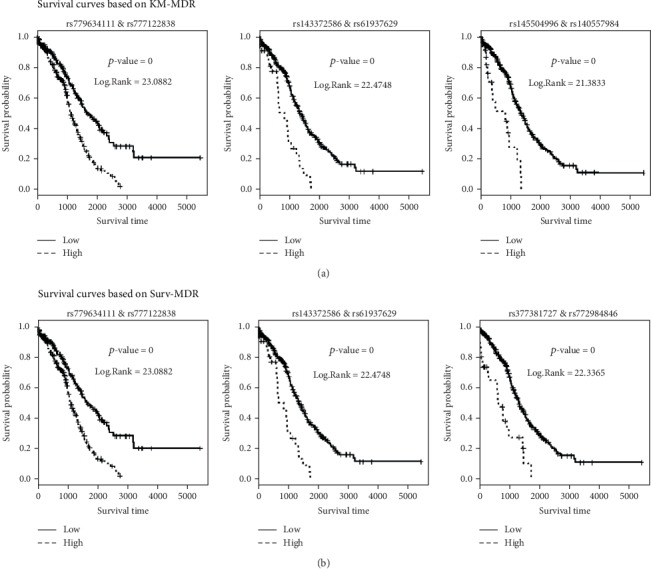
Survival curves for the high-risk group versus low-risk groups by the KM-MDR and Surv-MDR attribute of SNP pairs. (a) Survival curves based on KM-MDR. (b) Survival curves based on Surv-MDR.

Furthermore, we plotted the survival curves for high-risk versus low-risk groups defined by the KM-MDR and Surv-MDR attribute for SNP pairs listed in Tables 3 and 4. For a given pair of SNPs, we implemented KM-MDR (or Surv-MDR) to classify high- and low-risk groups for the training set (9/10 sets) and applied this classification rule to the corresponding testing set (1/10 sets). By repeating this procedure 10 times, all patients could be assigned into high- or low-risk groups by KM-MDR (or Surv-MDR), and the Kaplan-Meier survival curves for these two groups are plotted in Figure 4. Since the same procedure is implemented in calculating the testing score of the corresponding SNP pairs, the log-rank test statistic is the same as the testing score in Tables 3 and 4.

As shown in Figures 4(a) and 4(b), all six of the survival curves obtained by both KM-MDR and Surv-MDR for the attribute of SNP pairs are substantially separated, with a significant *p* value of the log-rank test. Since the 10-fold cross-validation process randomly divides the sample into 10 even groups, the results shown in Figure 4 could be a randomly chosen set of results. Nonetheless, the groups representing the six pairs of SNPs enable appropriate separation of the two survival curves and show significant associations with the survival time.

## 4. Discussion

In this paper, we propose a simple and computationally efficient method, KM-MDR, to identify GGIs associated with the survival phenotype. The KM-MDR method can be considered as an extension of QMDR to the survival phenotype by replacing the mean value of the quantitative trait with the Kaplan-Meier median survival time to classify multilocus genotypes into high- and low-risk groups. Since the survival time is commonly censored and often has a skewed distribution, the median survival time is a more useful and robust statistic for the central measure than the mean survival time in survival analysis. Therefore, it is natural to consider the median survival time as a new classifier to reduce high-dimensional genotypes into a binary attribute for the survival phenotype. In addition, the log-rank test statistic is most popularly used to compare the survival times between two groups, analogously to the *t* test in QMDR.

When comparing the process of KM-MDR with that of Surv-MDR, either the median survival time or a log-rank test statistic should be calculated nine times for two-way interaction model, because there are nine different genotypes available for two SNP pairs. As shown in the simulation results for the power comparison, the power of KM-MDR is almost the same as that of Surv-MDR for all cases. However, KM-MDR has the advantage of faster computation. Comparing the run time for one loop of the two-way interaction model, KM-MDR took 31.55 seconds and Surv-MDR took 37.49 seconds for the same procedure. In the analysis of ovarian cancer data, the KM-MDR method took 5.577 hours to search over all two-way models with 565 SNPs and 432 patients, while Surv-MDR took 6.003 hours. Thus, KM-MDR is computationally more efficient than Surv-MDR. Furthermore, the information of each cell is used once in KM-MDR, but it is used multiple times in Surv-MDR because the log-rank test compares the survival time between samples with and without the genotype, which may cause the distinction between the high- and low-risk groups to be contaminated. However, when only a few events are observed due to either a small size of the sample or heavy censoring, the classification for high- and low-risk groups may be not available in KM-MDR. In fact, there were a few cases in which a median survival time was not reached in the process of real data analysis with 10-fold cross-validation. Even if the sample is large enough, the median survival time may not be obtained when most patients are cured of a disease. In cases where overall median survival time is not available due to heavy censoring, KM-MDR cannot be applicable, whereas Surv-MDR has no such restriction. However, for the case that the median survival time of a specific cell is not obtained, we proposed an alternative method using complementary samples.

As shown in the simulation studies, KM-MDR has greater power than Cox-MDR and AFT-MDR when there is no covariate effect and seems to be robust even under heavy censoring, whereas the power of AFT-MDR decreases very rapidly when the censoring fraction becomes greater than 0.3. In addition, KM-MDR has moderate power even when there is a covariate effect and has a similar power to that of Cox-MDR as the censoring fraction increases. Although KM-MDR has the weakness of not being able to adjust for the covariate effect, it is at least as powerful Cox-MDR to detect GGIs for heavily censored survival data.

In this paper, we did not perform the simulation study for the higher-order interaction model due to heavy computation time for the higher-order model. However, we implemented the simulation study by considering only a 3-way interaction model which referred in [12]. We found that the power trend of all methods is similar to that shown in the 2-way interaction model though not given here.

In the analysis of a dataset of ovarian cancer patients from TCGA, the two methods showed similar results to those obtained in the simulation study. Four pairs of SNPs overlapped, and the testing scores were also similar over all pairs. In addition, all the permutation *p* values were very low, which implies that all of the top three pairs were significant interaction models. Furthermore, we plotted the survival curves between the high- and low-risk groups identified by the KM-MDR and Surv-MDR attributes of SNP pairs using 10-fold cross-validation. All survival curves showed a significant separation of the two groups in terms of the *p* value of the log-rank test.

## 5. Conclusion

We propose KM-MDR, a new extension of the MDR algorithm that enables an efficient identification of gene-gene interactions in a survival setting. Because it is simple and requires less computation, it is highly advantageous for dealing with high-dimensional biological data. We expect that KM-MDR will contribute to the identification of gene-gene interactions associated with numerous human diseases.

## Figures and Tables

**Table 1:** Type I error of KM-MDR.

MAF	*Cf* = 0	*Cf* = 0.1	*Cf* = 0.3	*Cf* = 0.5
0.05	0.017	0.024	0.035	0.028
0.10	0.029	0.025	0.025	0.031
0.20	0.025	0.015	0.029	0.032
0.30	0.026	0.029	0.031	0.029
0.40	0.028	0.022	0.024	0.020

MAF: minor allele frequency; Cf: censoring fraction.

**Table 2:** The 20 SNPs (out of 565 SNPs) with a main effect by fitting a Cox model.

SNP	Coefficients	*p* value	SNP	Coefficients	*p* value
rs372182118	-0.4443	0.0018	rs371397657	-0.3218	0.0304
rs142576028	-0.4463	0.0024	rs530110275	-0.3645	0.0308
rs147716822	-0.3879	0.0067	rs751038000	-0.3198	0.0320
rs143372586	0.4279	0.0074	rs76548941	0.3191	0.0323
rs143657395	0.3523	0.0133	rs187800837	-0.3060	0.0355
rs201622956	0.3353	0.0214	rs747796926	-0.2985	0.0374
rs745834619	-0.3325	0.0241	rs372938746	-0.3542	0.0386
rs80271292	0.3566	0.0274	rs142473318	-0.3029	0.0429
rs777282900	0.3128	0.0278	rs778818914	0.3147	0.0451
rs80039782	-0.3186	0.0301	rs760718931	-0.3145	0.0488

**Table 3:** Top three two-way models identified by KM-MDR.

With all 565 SNPs					With 545 SNPs excluding 20 SNPs with main effects				
Model	TRSC	TSSC	CVC	*p* value	Model	TRSC	TSSC	CVC	*p* value
rs145504996 rs140557984	19.6297	23.0881	1	0.001	rs779634111 rs777122838	19.6297	23.0881	1	0.000

rs143372586 rs61937629	16.7108	22.4748	1	0.000	rs145504996 rs140557984	15.2993	21.3833	1	0.000

rs779634111 rs777122838	15.2993	21.3833	1	0.000	rs201471889 rs150956058	16.5755	20.5237	1	0.001

TRSC: training score; TSSC: testing score; CVC: cross-validation consistency.

**Table 4:** Top three two-way models identified by Surv-MDR.

With all 565 SNPs					With 545 SNPs excluding 20 SNPs with main effects				
Model	TRSC	TSSC	CVC	*p* value	Model	TRSC	TSSC	CVC	*p* value
rs779634111 rs777122838	20.8030	23.0881	1	0.000	rs779634111 rs777122838	20.8030	23.0881	1	0.000

rs143372586 rs61937629	14.1778	22.4748	1	0.000	rs377381727 rs772984846	14.3477	22.3364	1	0.000

rs377381727 rs772984846	14.3477	22.3364	1	0.000	rs201471889 rs150956058	12.6174	20.5237	1	0.001

TRSC: training score; TSSC: testing score; CVC: cross-validation consistency.

## Data Availability

The real data used to support the findings of this study are available at https://gdc.xenahubs.net.
